# Local allergic rhinitis: entopy or spontaneous response?

**DOI:** 10.1186/s40413-016-0126-z

**Published:** 2016-12-06

**Authors:** Matteo Gelardi, Antonio V. N. Guglielmi, Lucia Iannuzzi, Vitaliano Nicola Quaranta, Nicola Quaranta, Massimo Landi, Mario Correale, Annamaria Sonnante, Margherita Rossini, Maria Addolorata Mariggiò, Giorgio Walter Canonica, Giovanni Passalacqua

**Affiliations:** 1 Section of Otolaryngology, Department of Basic Medical Science, Neuroscience and Sensory Organs, University of Bari, Bari, Italy; 2 School of Medicine, University of Bari, Bari, Italy; 3 National Paediatrics Healthcare, Turin, Italy; 4 Clinical Pathology, IRCCS S. De Bellis, Castellana Grotte, Bari, Italy; 5 Clinical Pathology, University of Bari, Bari, Italy; 6 Allergy and Respiratory Diseases, Department of Internal Medicine, IRCCS San Martino-IST-University of Genoa, Pad. Maragliano, Ospedale San Martino, L.go R. Benzi 10, Genova, 16133 Italy

**Keywords:** Allergic rhinitis, Nonallergic rhinitis, Sensitization, Nasal IgE, Local allergic rhinitis, Entopy

## Abstract

**Background:**

The existence of a local allergic rhintis was proposed on the basis of the detection of nasal IgE in the absence of a systemic sensitization. Nevertheless, the significance of this phenomenon remains still unclear.

We assessed the presence of mucosal nasal IgE in patients with ascertained allergic rhinitis, nonallergic rhinitis with inflammation and in healthy controls.

**Methods:**

Consecutive patients with a well ascertained diagnosis (clinical history, skin prick test, specific IgE assay, nasal endoscopy, nasal cytology) underwent an immunoenzymatic measurement of specific IgE to grass, cypress, parietaria and olive in nasal scrapings.

**Results:**

Fifteen patients with allergic rhinitis, 12 with non allergic rhinitis and 14 healthy subjects were studied. The patients with allergic and nonallergic rhinitis had higher nasal symptoms as compared to control subjects. Systemic sensitizatition (assessed by skin test and CAP-RAST) was obviously more frequent in allergic rhinitis, than in the other two groups. Allergen-specific nasal IgE could be detected in all groups (86,7, 33,3, and 50 % positive, respectively), even more frequently in the control group than in nonallergic rhinitis patients. No difference among allergens was identified. Out of the 26 non-allergic patients (non allergic rhinitis + controls) nasal IgE were positive in 11(42 %).

**Discussion:**

According to the results, the presence of nasal IgE against allergens seems to be a non-specific phenomenon, since they can be detected also in non allergic rhinitis and in healthy subjects.

**Conclusion:**

It can be hypothesized that the nasal IgE production represents a form of spontaneous immune response.

## Background

Rhinitis is defined as an inflammation of the nasal mucosa, usually characterized by rhinorrhoea, sneezing, nasal blockage and itching, variably associated. Those symptoms can be accompanied, expecially in the allergic form, by ocular symptoms such as lacrimation, eye itching, conjunctival hyperemia [1]. Allergic rhinitis (AR) is the most common form of non-infectious rhinitis, and it is triggered by an IgE-mediated immune response to allergens. Its prevalence reaches about 35 % [2] within the general population, and continues to increase [3].

Also non-allergic conditions can cause symptoms of rhinitis: infections, hormonal imbalance, physical/irritant agents, anatomical abnormalities or drugs [4]. Also the non-allergic form of rhinitis (NAR) are of clinical relevance. These are characterized essentially by an intense inflammatory infiltration, in the absence of a systemic IgE-sensitization. According to the predominant cell types, NAR can be distinguished into NARES (non-allergic rhinitis with eosinophils), NARMA (non-allergic rhinitis with mast cells), NARNE (non-allergic rhinitis with neutrophils) and NARESMA (non-allergic rhinitis with eosinophils and mast cells) [5–8].

Since 1975, another pathophysiological type of rhinitis was proposed, characterized by the presence of allergen-specific IgE only in the nose, without any evidence of systemic sensitisation detectable by skin prick test (SPT) or specific IgE serum assays (CAP-RAST) [9]. This observation suggested the term of “entopy”[10] and, subsequently, of “local allergic rhinitis” (LAR) [11]. These definitions are still a matter of debate, since LAR has not been yet clearly identified as an independent nosographic entity. With the aim of exploring more in depth the local aspects of IgE response, we evaluated the presence of nasal IgE, in two groups of patients with well identified forms of rhinitis, and in healthy controls.

## Methods

Adult patients were assessed for the presence of allergen-specific nasal IgE, after the type of rhinitis was classified in detail according to the diagnostic procedure. Patients were subdivided into AR (positive SPT and CAP-RAST), and NAR (negative SPT and CAP-RAST, with nasal inflammation). A control group of healthy subjects (no rhinitis symptoms and negative SPT/CAP-RAST) was also included for comparison. The study took place between November 2014 and January 2015 at the Rhinology Unit of the University of Bari. All subjects provided an informed consent for the management of their anonymous clinical data. The inner ethical committee was simply notified, since this was not an interventional study, no placebo was used, and the procedures were part of the standard diagnostic practice. According to the existing laws, also the healthy volunteers could be admitted, after written informed consent. The diagnostic work-up for rhinitis involved: personal clinical history, family history, symptoms (scores and visual analog scale), SPT, CAP-RAST assay, nasal endoscopy, nasal scraping for cytology, and assay for nasal mucosal IgE (see below). Those patients with symptoms of chronic rhinosinusitis, anatomical abnormalities (septal deviation, turbinate hyperthrophy), unilateral symptoms/signs or malignancies were excluded from the assessment of nasal IgE. We also excluded those patients reporting symptoms of possible acute infectious diseases (e.g. common cold) in the last month. All patients were medication-free (local/systemic antihistamines, local/systemic corticosteroids) in the past 2 weeks.

### Clinical assessment

Obstruction and itching were assessed by a 10-cm visual analog scale (0 = totally obstructed to 10 = absent; 0 = absent to 10 = intense, respectively) [12]. Rhinorrhea and sneezing were simply assessed as present/absent. Positive family history was defined as the presence of rhinitis and/or asthma in at least one parent.

### Skin prick test

It was performed using a panel of the most common aeroallergens (Stallergenes, Milan, Italy) according to the recommendations of the European Academy of Allergy and Clinical Immunology: house dust mite, grass mix, *Parietaria*, olive, cypress, mugwort, alternaria, ragweed, cat and dog dander [13].

### Serum IgE assay (CAP-RAST)

Allergen-specific IgE antibodies against the same allergens assayed with skin test were measured by a quantitative immunoassay (Immunocap® Thermo Fisher Scientific Inc. Uppsala, Sweden). The measure range of the test varies from 0.1 to 100 kU/L, and in clinical practice, 0.35 kU/L is commonly used as a the optimal lower cut-off.

### Nasal endoscopy

It was carried out by a 3,4 mm diameter flexibile-fibroscope (Vision-Sciences® ENT-2000), to assess the presence of major abnormalities, such as septal deviation, polyposis, turbinate hypertrophy, or exudation from the ostiomeatal complex.

### Nasal cytology

This procedure was performed by scraping the middle part of the inferior turbinate with a Rhino-Probe® device (Arlington Scientific). The sample was smeared on a slide, air-dried, then stained with the May-Grünwald Giemsa preparation. The type and cell number were examined using microscopy (Nikon® E600). Cell types were identified, and intracellular components were studied at x1000 in oil immersion. The mean number per 50 fields was calculated and reported [14, 15]. This noninvasive method allows to obtain representative samples of the nasal mucosa and its cellular components [16].

### Nasal IgE

Samples for nasal IgE assay were collected by Rhino-probe® scraping, as for nasal cytology. Samples were diluted in 0.5 mL physiological solution, then stored at 4 °C. Measurement of nasal specific IgE was carried out by the Immunocap® Specific IgE tests (Thermo Fisher Scientific Inc, Uppsala, Sweden) by a quantitative immunoassay. The test is the same routinely used on serum or plasma samples. In order to establish a lower threshold limit of detection we tested 10 negative controls (healthy, negative SPT and CAP-RAST), and the limit resulted to be 0.17 kU/L. Specific IgE to parietaria, olive, dust mite, cypress and grasses were assayed.

### Statistical analysis

Demographic and clinical characteristics were analyzed by descriptive statistics (% or mean and SD). Continuous variables were analyzed by the t test for independent samples, whereas the Chi-square test was used for qualitative measures. The 3 groups were compared by the ANOVA test. P values for the null hypothesis was set at 0.05. The dedicated software SPSS22 was employed.

## Results

**Fig. 1:**
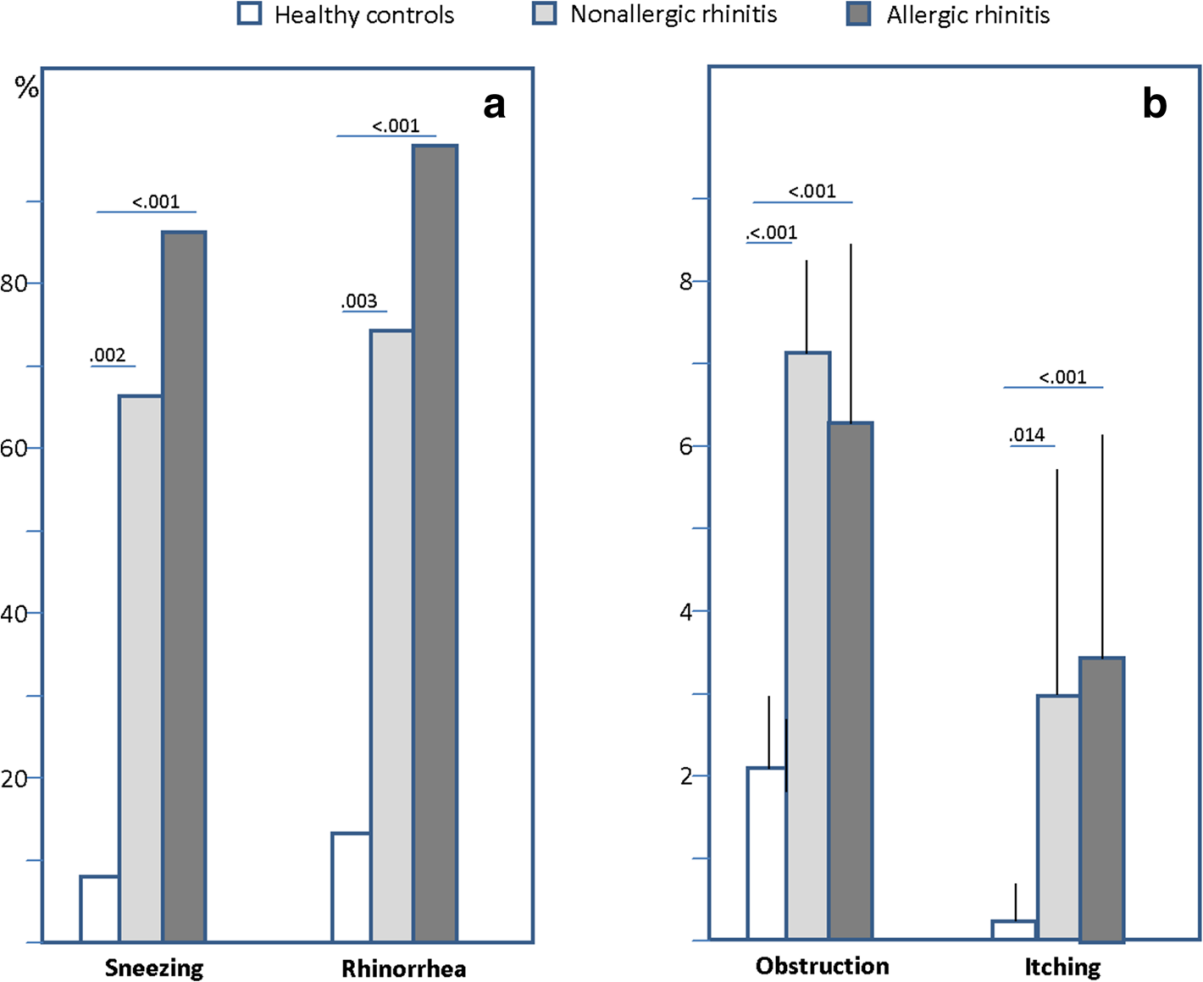
Panel **a** (*left*): % of patients reporting symptoms of sneezing and rhinorrhea. Panel **b** (*right*) visual analog scale score for obstruction and itching (mean and SD). Significant p values are reported above the bars

**Fig. 2:**
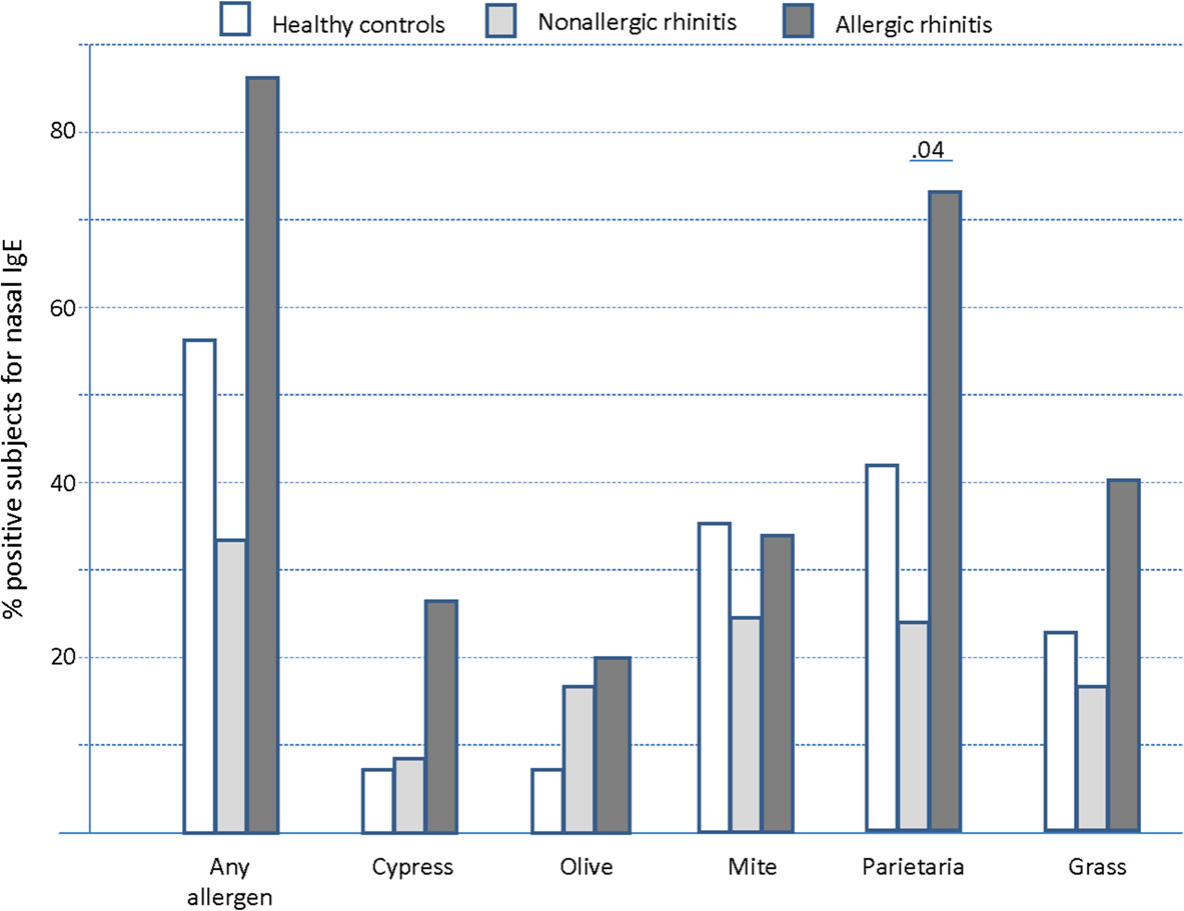
% of patients with positive assay for nasal IgE, in total and for each single allergen. A significant difference among the 3 groups was detected only for Parietaria. Significant p values are reported above the bars

Fourty-one patients (19 male, mean age 36.2 ± 16.3 years) were studied between November 2014 and January 2015. All the subjects were consecutively enrolled for the nasal IgE assay, once a diagnosis was made on the basis of the procedures mentioned before. The studied population was subdivided into: AR (*n* = 15), NAR (*n* = 12) and controls (*n* = 14). These latter included volunteers enrolled among healthcare professionals, postgraduate students or nurses. The 3 groups were homogeneous for demographic characteristics as summarized in Table 1. Unexpectedly, a positive family history for atopy was more frequent in the NAR group. Those patients with AR or NAR had significantly more severe rhinorrhea, itching, sneezing and obstruction versus the control subjects (Fig. 1). As per inclusion criteria, only AR patients had positive IgE assay and SPT, as detailed in Table 1. The distribution of serum and SPT positivities for each allergen was consistent (Table 2), although the positivity for nasal IgE was, on average, lower with respect to the standard diagnostics. In particular, the serum IgE assay provided more frequently positive results, although the difference among groups was not significant. The mean values of nasal IgE (irrespectively of the allergen) in AR, NAR and controls were 1.03 ± 0.84, 0.79 ± 0.90 and 0.67 ± 0.45 kU/L, respectively. Looking more in depth to nasal IgE, (cypress, olive, dust mite, parietaria, grass), there was no significant difference in the number of patients proving positive among AR, NAR and controls (86, 33.3, and 57.1 %, respectively), but the control group had an overall higher rate of local IgE positivity versus the NAR group, although this difference was not statistically significant (Fig. 2 **).** More in detail, it appeared that in the AR group there was a higher occurrence of positivities vs NAR and controls for cypress (26,7 % vs 8,3 % vs 7,1 %), olive (20 % vs 16.7 % vs 7.1 %), and grass (40 % vs 16.7 % vs 21.4 %). Local nasal IgE to Parietaria were detected more frequently in the AR patients than in NAR and controls (73.3 % vs 25 % vs 42.9 %; *p* = 0.04) (Fig. 2 **)**. Among non allergic patients (NAR + controls, *N* = 26) nasal IgE were detectable for parietaria (67.9 %), olive (26.8 %,), cypress (15.4 %), dust mite (60.7 %) and grasses (38.1 %). There was no significant difference concerning each single allergen, except for Parietaria, more frequently positive in AR patients. As expected, there was a significant difference in the differential cell count at nasal cytology between the patients with and without rhinitis. This was true for each cell type (Table 3). No difference in the percentage of degranulating mast cells was found between AR and NAR patients.

**Table 1:** Demographic, clinical and sensitization characteristics of the patients

	HEALTHY CONTROLS (*n* = 14)	NONALLERGIC RHINTIS (*n* = 12)	ALLERGIC RHINITIS (*n* = 15)	*p*
Age, mean ± SD	37.6 ± 16.3	32.4 ± 15.2	37.8 ± 17.7	0650
Sex (M/F)	8/6	4/8	10/5	0214
ASTHMA n (%)	1 (7.1)	2 (16.7)	3 (21)	0.1
Family history, n (%)	0	4 (33.3)	1 (7.1)	0028
ASA sensitivity, n	0	0	0	1,0
SPT positive, n (%)	0	0	15 (100)	0.001
Grass	0	0	3 (20)	
Parietaria	0	0	2 (13)	
Olive	0	0	4 (26)	
Cypress	0	0	6 (40)	
Dust mite	0	0	5 (33)	
CAP-RAST positive n(%)	0	0	15 (100)	0.001
Grass	0	0	6 (40)	
Parietaria	0	0	8 (54)	
Olive	0	0	7 (47)	
Cypress	0	0	10 (67)	
Dust mite	0	0	8 (54)

**Table 2:** N and % of positive patients to SPT, CAP-RAST and nasal IgE assay in AR patients (*N* = 15)

	SPT+	CAP-RAST+	Nasal IgE+
Cypress	6 (40)	10 (67)	4 (27)
Olive	4(26)	7(47)	3 (20)
Dust Mite	5 (33)	8 (53)	5 (33)
Parietaria	2 (13)	8 (53)	11 (73)
Grass	3 (20)	6(40)	6 (40)

**Table 3:** Results of nasal cytology in the three studied groups

	HEALTHY CONTROLS (*n* = 14)	NONALLERGIC RHINITIS (*n* = 12)	ALLERGIC RHINITIS (*n* = 15)	*P* value ^a^
Neutrophils	23,5 ± 5,8	303,4 ± 19,3	502,5 ± 24,4	<0.001
Eosinophils	0	82,5 ± 10,4	59,4 ± 7,9	<0.001
Lymphocytes	7,3 ± 2,3	8,7 ± 3,9	34,5 ± 5,9	<0.001
Mast cells	0	28,5 ± 6,3	10,3 ± 3,7	<0.001

^a^Allergic Rhinitis vs the other groups (Non Allergic Rhinitis and Controls)

## Discussion

In general, more than 60 % of patients with NAR receive a generic diagnosis of “idiopathic” rhinitis, that is based on an exclusion criterion [8]. Although the pathophysiology of those forms of rhinitis remains poorly defined, an infiltration of eosinophils, mast-cells and T-lymphoctyes can be frequently evidenced, this supporting the presence of an active inflammation. Based on this, the hypothesis of a “local” allergic reaction has been suggested.

In 1975 Huggings demonstrated, in a qualitative way, the presence of house dust mite-specific IgE in the nose of subjects with symptoms of rhinitis but no evidence of systemic IgE [9], whereas in 2003 Powe introduced the concept of “entopy” [10]. He assessed 32 patients [11 with AR, 10 with idiopathic rhinitis and 12 controls), and found nasal grass-specific IgE in 3 of the patients with idiopathic rhinitis. Thus the term entopy was used to indicate an allergy confined to the nasal mucosa, in the absence of the evidence of systemic sensitization. Subsequently, in 2004, the strict immunological interaction between T lymphocytes and mast cells in entopy were described [17, 18]. Other authors, evaluating relatively large groups of patients, introduced the term of “local allergic rhinitis” (LAR), to describe the isolated presence of specific IgE in the nasal mucosa. In this regard, the specific nasal provocation test was used to diagnose the disease [11, 19].

Our results confirmed the presence of nasal IgE in AR subjects, usually in agreement with the systemic sensitization profile, revealed by IgE serum assay and SPT. Interestingly, nasal specific IgE could be detected also in a relevant percentage of patients with NAR. More importantly, as unexpected finding, nasal allergen-specific IgE could be found also in 50 % of the healthy control subjects. Although the number of patients studied is small, the percentage remains highly significant. This latter aspect would suggest, as an hypothesis, that a local secretion of IgE could be part of a spontaneous immune response to environmental agents. The identification of nasal allergen-specific IgE also in healthy subjects and in patients with non allergic rhinitis suggest that the concept of LAR should be reconsidered and re-evaluated. These observations also suggest that a more detailed diagnostic approach, involving nasal endoscopy and nasal cytology, should be carried out when the diagnosis is uncertain. In fact, nasal cytology can better define and refine the details of nasal inflammation. The technique is not invasive, does not alter the cellular profile and it is also used in electron microscopy studies for other diseaes [16, 20]. According to this, in the presence of symptoms of rhinitis, without a clear evidence of an allergic sensitization, the detection of mucosal nasal IgE should not be considered as a certain demonstration of LAR. In addition, NAR cannot be immediately defined as LAR, even in the presence of local IgE, because of both severity, cytological pattern and, possibly, the association with polyposis [21]. It can be argued that the aforementioned studies on LAR suffered from a relatively low specificity. The allergen nasal provocation test implies the administration of allergens at high concentration, that can provoke a non specific response expecially in NAR where the nasal mucosa is already inflamed [22]. Also, acoustic rhinometry is now considered poorly reproducible and difficult to standardize [23]. In this regard, the active anterior rhinomanometry after decongestion would be preferable from a functional point of view [24, 25]. To better define the pathophysiological picture, an incremental-concentration nasal specific challenge with each allergen should be performed in NAR and controls, but this was not part of the aims of the present work.

Indeed, the postivity of allergen-specific nasal IgE in the absence of a detectable systemic sensitization remains unclear, needs more experimental proof [26, 27], and is anyway not explained by this experimental study. In fact the main limitation of the present study is the absence of specific nasal provocation tests. On the other hand, the presence of nasal allergen specific IgE in heathy subjects (without symptoms, but exposed to allergens) would not justify the use of a challenge. We can only hypothesize that the nasal mucosa, exposed since birth to allergens, can evoke an IgE synthesis in the context of the immune response, independently of the atopic sensitization [28]. This is indirectly confirmed by the fact that in allergic patients the presence of local nasal and systemic IgE to allergens are consistent, expecially for perennial allergens (parietaria 34,6 % and mites 26 %. Finally, to date there is no formal evidence that LAR can evolve into an AR with a systemic IgE production [29].

## Conclusion

The detectable presence of allergen-specific IgE on the nasal mucosa of subjects with nonallergic rhinitis and healthy subjects suggests that this phenomenon could be the expression of an innate response, rather than a specific local reactivity.

## Abbreviations

AR: Allergic rhinitis
LAR: Local allergic rhinitis, SPT, Skin Prick Test
NAR: Nonallergic rhinitis
NARES: Non-allergic rhinitis with eosinophils
NARESMA: Non-allergic rhinitis with eosinophils and mast cells
NARMA: Non-allergic rhinitis with mast cells
NARNE: Non-allergic rhinitis with neutrophils
